# In vivo cation exchange in quantum dots for tumor-specific imaging

**DOI:** 10.1038/s41467-017-00153-y

**Published:** 2017-08-24

**Authors:** Xiangyou Liu, Gary B. Braun, Mingde Qin, Erkki Ruoslahti, Kazuki N. Sugahara

**Affiliations:** 1 Cancer Research Center, Sanford Burnham Prebys Medical Discovery Institute, 10901 N Torrey Pines Road, La Jolla, California 92037 USA; 2 Department of Surgery, Herbert Irving Comprehensive Cancer Center, Columbia University College of Physicians and Surgeons, 1130 St Nicholas Avenue, New York, New York 10032 USA; 3 Program of Materials Science and Engineering, University of California, San Diego, 9500 Gilman Drive, La Jolla, California 92093 USA; 4 Center for Nanomedicine and Department of Cell, Molecular and Developmental Biology, University of California, Santa Barbara, Santa Barbara, California 93106 USA

## Abstract

In vivo tumor imaging with nanoprobes suffers from poor tumor specificity. Here, we introduce a nanosystem, which allows selective background quenching to gain exceptionally tumor-specific signals. The system uses near-infrared quantum dots and a membrane-impermeable etchant, which serves as a cation donor. The etchant rapidly quenches the quantum dots through cation exchange (ionic etching), and facilitates renal clearance of metal ions released from the quantum dots. The quantum dots are intravenously delivered into orthotopic breast and pancreas tumors in mice by using the tumor-penetrating iRGD peptide. Subsequent etching quenches excess quantum dots, leaving a highly tumor-specific signal provided by the intact quantum dots remaining in the extravascular tumor cells and fibroblasts. No toxicity is noted. The system also facilitates the detection of peritoneal tumors with high specificity upon intraperitoneal tumor targeting and selective etching of excess untargeted quantum dots. In vivo cation exchange may be a promising strategy to enhance specificity of tumor imaging.

## Abstract (web summary)

The imaging of tumors in vivo using nanoprobes has been challenging due to the lack of sufficient tumor specificity. Here, the authors develop a tumor-specific quantum dot system that permits in vivo cation exchange to achieve selective background quenching and high tumor-specific imaging.

## Introduction

The design of nanoprobes for in vivo tumor imaging has traditionally focused on optimization of probe properties such as size, surface coating, and signal intensity to maximize target sensitivity and specificity^1, 2^. Nanoparticles larger than the renal filtration threshold (~ 6 nm) circulate longer and accumulate more efficiently in tumors than small molecules. However, long washout periods increase background signals especially in the mononuclear phagocyte system (MPS; e.g., liver, spleen). Although surface modification with polyethylene glycol (PEG) reduces non-specific uptake by liver Kupffer cells, the tumor to liver ratio (T/Li) for nanoparticles that are not cleared through the kidneys generally decreases with time, leading to degraded image contrast^3–5^. In this study, we explore an alternative strategy to enhance tumor specificity—selective elimination of background signals while preserving tumor signals in vivo. We utilize photoluminescent quantum dots (QDs) as the platform for quenchable nanoprobes based on the ability of QDs to undergo cation exchange (ionic etching) with external metal ions.

Cation exchange in QDs allows rapid modification of the elemental composition and crystal structure, and has been exploited to synthesize new nanostructures and modify photoluminescence (PL) characteristics^6, 7^. In QD cation exchange, metal cations that are embedded within an anion lattice can exchange with free metal ions in solution. In particular, in QDs built from large polarizable sulfide (S^2−^), selenide (Se^2−^), or phosphorus (P^3−^), the internal cations can pass through open sites between anions leading to effective cation exchange. Notably, the anionic framework and geometry of the QD core may be preserved during cation exchange^6^.

Here, we introduce a biocompatible QD platform, which loses PL upon cation exchange, and achieves tumor-specific in vivo imaging in 3 steps: First, active delivery of the QDs into extravascular tumor tissue and cells to gain bright tumor signals. Second, induction of cation exchange in excess QDs remaining in the circulation to quench background signals. Third, effective renal excretion of the cations released from the QDs to minimize potential toxicity.The platform also probes peritoneal tumors with high specificity when delivered through the abdominal cavity suggesting its potential role as an aid in the diagnosis and surgery for peritoneal carcinomatosis.

## Results

### Synthesis of PEGylated near-infrared QDs

**Fig. 1:**
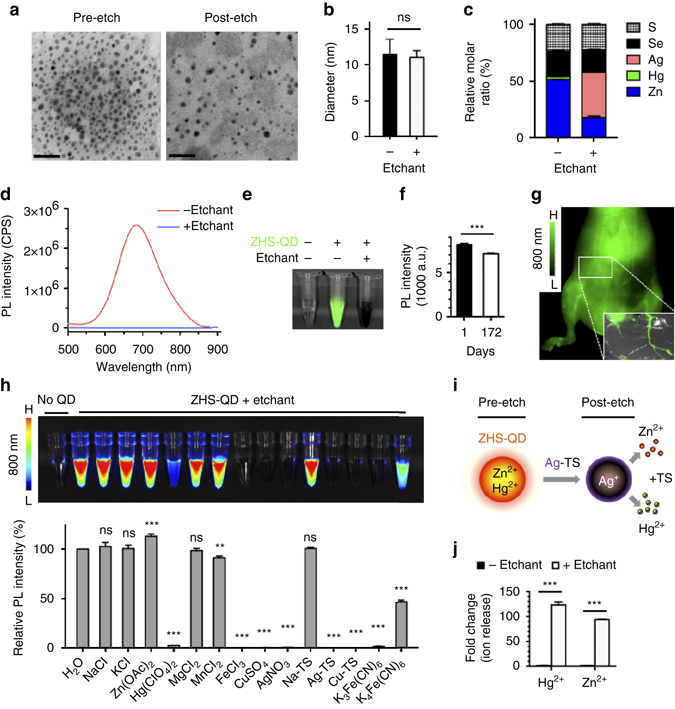
Characterization of etchable ZHS-QDs. **a**, **b**, **d** TEM images **a** hydrodynamic diameters **b** and PL spectra at 450 nm excitation **d** of ZHS-QDs before and after Ag-TS treatment. *n* = 6 per group; *Scale bars*, 50 nm. **c** EDS analysis of ZHS-QDs before and after Ag-TS treatment. Refer to Supplemental Table 1 for complete data set. *n* = 3 per group. **e** NIR image of ZHS-QDs (green) with and without Ag-TS treatment taken with a Li-Cor Pearl imager under an 800 nm channel. **f** NIR signals of ZHS-QDs quantified at days 1 and 172 post-synthesis. *n* = 5 per group. **g** NIR images of the ventral side of a mouse intravenously injected with ZHS-QDs. Inset, ex vivo NIR image of the skin. **h** In vitro etching of ZHS-QDs by various chemicals. NIR images (*upper panel*) and the emission intensity of the ZHS-QDs before and after etching (*bottom panel*, *n* = 4 per group) are shown. Each column corresponds to the tube above. **i** Schematic of ZHS-QD etching. Ag-TS quenches ZHS-QDs by providing Ag^+^ in exchange with Zn^2+^ and Hg^2+^. **j** Hg and Zn released from ZHS-QDs with and without Ag-TS treatment. *Columns* represent fold over non-etched group. *n* = 3 per group. Statistics, Student’s *t*-test **b**, **f**, **j** or one-way analysis of variance **h**; *error bars*, SEM; ns, not significant; **P < 0.01; ***P < 0.001

We first synthesized highly dispersed near-infrared (NIR) Zn_*x*_Hg_1-*x*_Se_*y*_S_1-*y*_ QDs (ZHS-QDs) consisting of zinc (Zn^2+^), mercury (Hg^2+^), Se^2−^ and S^2−^. Hg^2+^ was doped into the core as a tracer to accurately study tissue distribution and clearance kinetics. The QDs were coated with PEG to reduce MPS uptake^4^. Transmission electron microscopy (TEM) revealed a core diameter of 6.6 ± 2.3 nm (mean ± standard deviation; Fig. 1a and Supplementary Fig. 1a). Dynamic light scattering (DLS) showed a hydrodynamic diameter of ~ 12 nm (Fig. 1b), a size above the renal filtration threshold^8^.Elemental analysis by inductively coupled plasma optical emission spectroscopy (ICP-OES) and energy-dispersive X-ray spectroscopy (EDS) confirmed the composition of the QDs (Fig. 1c and Supplementary Fig. 1b). PEG was detected by EDS as carbon (C) and oxygen (O), which accounted for ~ 90% of the total atoms (Supplementary Table 1). The PL emission peak was at 685 nm, which was consistent under different excitation wavelengths (Fig. 1d and Supplementary Fig. 1c). The quantum yield (QY) was 12% based on a calculation using Rhodamine 6G as a standard. A strong PL signal at 800 nm(the tail of the emission peak) was obtained using 785-nm excitation (Fig. 1e and Supplementary Fig. 1d), the preferred excitation wavelength for a Li-Cor Pearl Impulse imager to minimize background signals during in vivo imaging (Supplementary Fig. 1e). Approximately 90% of the PL intensity remained after the QDs were stored at 4 °C for 6 months without visible aggregation of the QDs (Fig. 1f). Intravenous injection of the ZHS-QDs provided strong NIR vascular signals in mice (Fig. 1g). Additional QD properties are summarized in Supplementary Table 2.

### In vitro cation exchange reactions in ZHS-QDs

We next developed an ‘etchant’, which serves as a cation donor to the QDs and quenches the PL of the QDs by cation exchange. Various chemicals with cation-exchange capacity quenched ZHS-QDs in vitro except for Zn(OAc)_2_ (zinc acetate dehydrate), which increased the PL probably by providing extra Zn^2+^ to the QDs (Fig. 1h). One of the most efficient etchants was Ag(S_2_O_3_)_2_^3−^ (Ag-TS), which consisted of silver ions (Ag^+^) stabilized with thiosulfate (TS), a metal chelator used as a clinical detoxifying agent for heavy metal ions^9, 10^. Ag-TS provided Ag^+^to the QDs in exchange with Zn^2+^ and Hg^2+^ (Fig. 1i), and rapidly quenched the QDs (Fig. 1d,e). Stoichiometric in vitro quenching studies in aqueous solution at room temperature showed that > 95% of ZHS-QDs were quenched when Ag-TS was added at an Ag to Zn molar ratio of 1:1 or higher (Supplementary Fig. 1f). Treating ZHS-QDs with Ag-TS (Ag to Zn = 1:1) resulted in near complete quenching of the QDs within 10 s (Supplementary Fig. 1g). These results are consistent with earlier studies, which reported rapid and nearly complete cation exchange reactions under ambient conditions^6, 11^. The completion of cation exchange can be attributed to several factors: adequate Ag to Zn molar ratio, the small number of atomic layers and large surface area of QDs, the high thermodynamic driving force to form the exchanged product (Ag_2_S and Ag_2_Se), and the TS solubilizing agent, which ligates free Ag^+^, Zn^2+^ and Hg^2+^ ions to minimize the formation of any surface nanoprecipitate that might otherwise reduce exchange rates^7^.

ICP-OES and EDS of the QDs showed that Ag-TS treatment led to the release of Hg^2+^and Zn^2+^ from the core in exchange for Ag^+^ (Fig. 1c and Supplementary Fig. 1b). The released Hg^2+^ and Zn^2+^ were detected in the buffer by ICP-OES (Fig. 1j). These results confirmed that effective cation exchange was achieved. More than 80% of the C and O remained after Ag-TS treatment suggesting minimal change in the surface PEG layer (Supplementary Table 1). The XRD spectra of ZHS-QDs after Ag-TS treatment showed that new peaks corresponding to Ag_2_S and Ag_2_Se appeared confirming the ICP-OES and EDS results (Supplementary Fig. 1h). Accordingly, the extinction coefficient of the QDs increased after etching, consistent with Ag-TS inducing a material transformation of the core (Supplementary Table 2). The QD core size after etching was 6.0 ± 1.9 nm suggesting a small change in the size of the QDs (Fig. 1a and Supplementary Fig. 1a). The hydrodynamic size also remained unchanged and precipitation was not observed (Fig. 1b).

### In vitro quenching of various QDs induced by Ag-TS

**Fig. 2:**
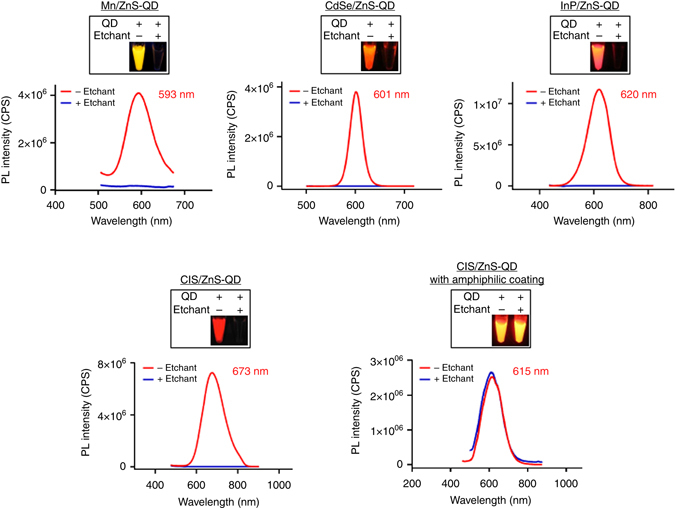
In vitro etching of various QDs with Ag-TS. PL spectra of the QDs before (red) and after (blue) etching are shown. The emission peaks of the QDs are also listed. The boxed panels show fluorescence images of the QD aqueous solutions with or without etching. QDs synthesized in-house (i.e., Mn/ZnS-QD) and high-quality commercial QDs (i.e., CdSe/ZnS-QD, InP/ZnS-QD, and CIS/ZnS-QD) were each etchable by Ag-TS. The Mn/ZnS-QD, InP/ZnS- and CIS/ZnS-QDs had coatings of 3-mercaptopropionic acid. Amphiphiliccoating with HS-(CH_2_)_11_-(OCH_2_CH_2_)_6_-NH_2_ prevented CIS/ZnS-QD from being etched

Ag-TS quenched QDs with differences in PL emission peak, QY, and composition, such as manganese-doped zinc sulfide (Mn/ZnS) QDs(QY = 7%)^12^,cadmium selenide zinc sulfide (CdSe/ZnS) core/shell QDs(QY = 45%), indium phosphide zinc sulfide (InP/ZnS) QDs(QY = 40%), and copper indium sulfide zinc sulfide (CIS/ZnS) QDs (QY = 15%; Fig. 2). Of note, amphiphilic coating with HS-(CH_2_)_11_-(OCH_2_CH_2_)_6_-NH_2_ made CIS/ZnS-QDs resistant to Ag-TS treatment likely because the coating inhibited cation penetration. These results suggest the wide applicability of chemical etching to various types of QDs, and a way to produce etching-resistant QDs. In addition, combination of QDs with different colors and etchable properties may allow multicolor and multiplex in vivo imaging.

### In vivo cation exchange reactions in ZHS-QDs

**Fig. 3:**
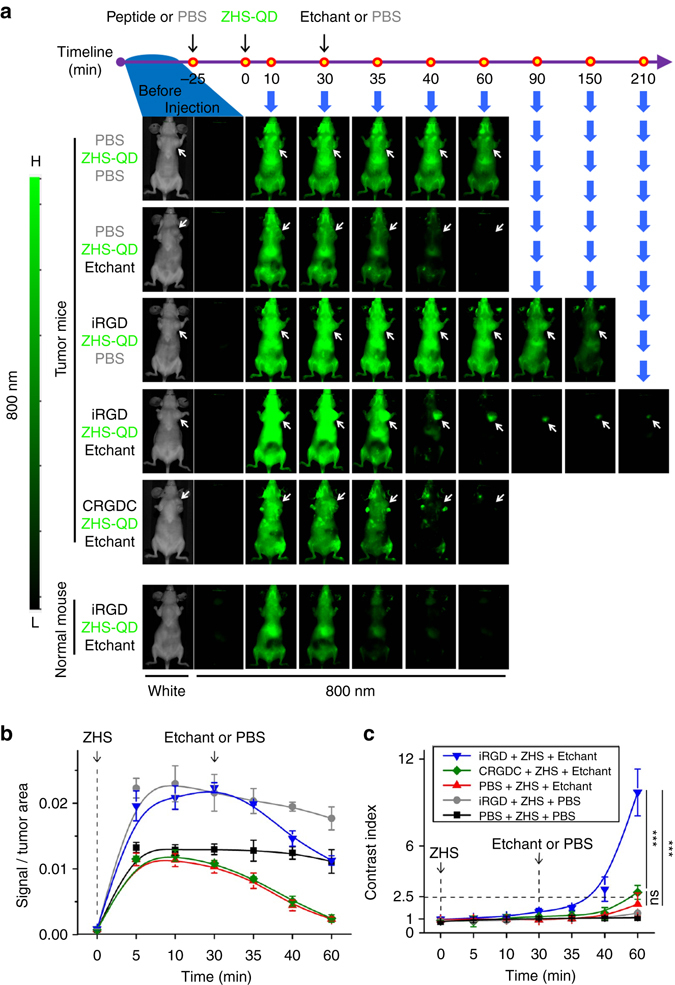
Time-dependent in vivo breast tumor imaging with etchable ZHS-QDs. Normal mice and mice bearing orthotopic MCF10CA1a human breast tumors received an intravenous injection of iRGD, CRGDC, or PBS before intravenous ZHS-QD injection. Ag-TS or PBS was given intraperitoneally. *n* = 4 per group. **a** The mice were anesthetized and imaged from the ventral side with a Li-Cor Pearl imager under 800 nm channel at the indicated time points. *Arrows*, tumors. **b** NIR signals in the tumor per unit area plotted against time. **c** Time-dependent changes of CI in the tumor area. Statistics, two-way analysis of variance; *error bars*, SEM; ns, not significant; ***P < 0.001

Intravenous injection of Ag-TS into mice eliminated systemic ZHS-QD signals within 30 s (Supplementary Fig. 2a). The etchant was quickly excreted or functionally consumed because a second dose of ZHS-QDs 10 min later produced strong signals. A second dose of Ag-TS effectively quenched the QDs indicating that repetitive in vivo imaging can be performed over short intervals. Intraperitoneal injection of Ag-TS completely eliminated QD signals in ~ 30 min reflecting the absorption kinetics of intraperitoneal Ag-TS into the blood stream^13^(Fig. 3a and Supplementary Fig. 2b). Delaying the timing of intraperitoneal Ag-TS injection allowed the QDs to enter the liver in higher quantity, and led to a significant liver signal after etching (Supplementary Fig. 2c,d). The resistance of the liver signals to Ag-TS treatment suggested that some QDs were phagocytosed by Kupffer cells, which protected the QDs from the circulating etchant. The liver signals became more evident when we modified the surface of ZHS-QDs with positive charge, which makes nanoparticles more prone to uptake by Kupffer cells^14^ (Supplementary Fig. 2e). Subcutaneous etchant injections were also effective. Three consecutive subcutaneous injections of a mixture of penicillamine, a clinical metal chelator^15, 16^, and CuSO_4_, which efficiently etched ZHS-QDs in vitro (Fig. 1h), led to near-complete in vivo quenching of systemic ZHS-QD signals in 20 min (Supplementary Fig. 2f).

Elemental analysis of biological samples collected from mice that received an intravenous ZHS-QD injection revealed progressive Hg^2+^ shift from the blood into the liver and spleen, indicating MPS uptake of the QDs (Supplementary Fig. 3a). Subsequent etching with intraperitoneal Ag-TS caused rapid redirection of Hg^2+^ into the kidney and urine, apparently as a result of in vivo cation exchange and renal clearance of Hg^2+^. The liver Hg^2+^ level in the etched group remained as high as that in the non-etched group up to 24 h likely reflecting the QDs that had already entered the liver by that point combined with uptake of free Hg^2+^ (a product of etching) by hepatocytes^17^. However, the level gradually decreased overtime in contradiction to the progressive increase observed in the non-etched group. Hg^2+^ became nearly undetectable in all the organs tested within 10 days of etching. ICP-OES analysis of ZHS-QDs isolated from the serum of the mice showed that the Ag-TS treatment caused a decrease in Zn, near complete elimination of Hg, and new incorporation of Ag in the QDs supporting the notion that in vivo cation exchange occurred (Supplementary Fig. 3b). Biochemical analysis of mouse blood samples 24 h and 1 week after ZHS-QD and/or Ag-TS injections did not show elevated transaminases or hyperbilirubinemia suggesting minimal hepatotoxicity (Supplementary Fig. 3c). ZHS-QDs did not cause renal impairment. A possible transient creatinine increase was associated with Ag-TS injections, but it was statistically insignificant. No microscopic pathological changes were found (Supplementary Fig. 3d).

### Passive distribution of ZHS-QDs into tumors

Circulating nanoparticles can passively distribute into tumors by enhanced permeability and retention (EPR) effect^18^. However, this process requires several hours, and passive distribution may not be enough to deliver compounds in sufficient quantity against high interstitial fluid pressure in tumors^18, 19^. In our experiments, intravenous injection of PBS and ZHS-QDs followed by intraperitoneal injection of Ag-TS resulted in minimal tumor signals in mice bearing orthotopic MCF10CA1a human breast cancer xenograft tumors (Fig. 3a).

### Tumor-specific systemic delivery of etchable ZHS-QDs

To actively deliver ZHS-QDs to tumors, we used the tumor-penetrating cyclic peptide, iRGD (amino acid sequence: CRGDKGPDC)^20^. iRGD carries a tumor-specific RGD motif and an RXXR/K motif (CendR motif). The RGD motif initially targets the peptide to tumor vasculature by binding to αv integrins, and after a proteolytic cleavage step, the CendR motif interacts with neuropilin-1. The interaction with neuropilin-1 increases extravasation and energy-dependent endocytosis of the peptide and bystander molecules in a tumor-specific manner^20–22^. The bystander effect peaks in about 30 min and remains for up to 60 min, providing a window to deliver co-injected free compounds into the extravascular tissue in a tumor-specific manner^23^.

ZHS-QDs were intravenously injected into MCF10CA1a breast tumor mice 25 min after an intravenous dose of iRGD, which led to a strong tumor signal buried in systemic background signals (Fig. 3a). The tumor signal became more visible as the background gradually faded over time likely because of QD clearance by MPS or quenching by various endogenous factors such as oxidation^24–26^. However, the signal to noise ratio remained low (Fig. 3c). In contrast, etching with intraperitoneal Ag-TS performed 30 min after ZHS-QD injection steeply lowered the background signals making the tumor-specific signal become apparent (Fig. 3a). The background signals decreased to ~ 10% within 10 min from etching, while 75% of the tumor signal remained (Fig. 3b). After 30 min, almost no background was noted, while over 50% of the tumor signal remained. The tumor-specific signal stayed apparent for at least 210 min after etching. CRGDC, a control RGD peptide without tissue-penetrating properties^20, 27^, did not enhance tumor signals. Etching decreased tumor signals to a similar degree in each group indicating baseline passive diffusion of Ag-TS into the tumors regardless of peptide pre-injection.

Contrast index (CI), defined as equation (1), is a parameter for tumor-specific image quality^3, 28^.

1 $$\begin{array}{*{20}{l}}{\rm{CI}} = \frac{{{\rm{fluorescence}}\,{\rm{intensity}}\,{\rm{of}}\,{\rm{tumor}}\,{\rm{area}} - {\rm{autofluorescence}}}}{{{\rm{fluorescence}}\,{\rm{intensity}}\,{\rm{of}}\,{\rm{normal}}\,{\rm{contralateral}}\,{\rm{region}} - {\rm{autofluorescence}}}} \hfill \end{array}$$

A CI of 2.5 is a general cut-off for substantial tumor-specific detection with optical imaging^3^. In breast tumor mice that received iRGD, ZHS-QDs, and Ag-TS, CI reached 2.5 at 10 min post-etching, followed by a continuous increase to reach 10 after an additional 20 min (Fig. 3c). Mice that received PBS or CRGDC pre-injection instead of iRGD reached a CI of ~ 2.5 after etching, representing the EPR effect. However, the dim tumor signals were suboptimal for practical imaging.

**Fig. 4:**
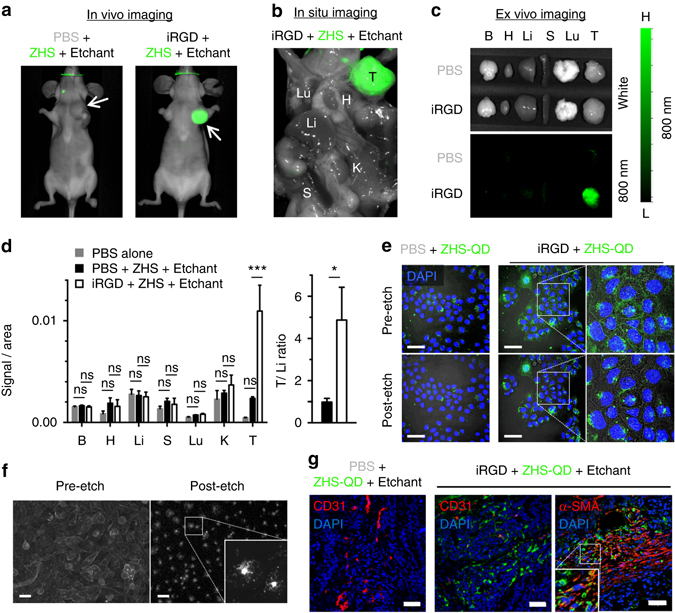
Breast tumor-specific imaging achieved by extravascular delivery of etchable ZHS-QDs. Mice bearing orthotopic MCF10CA1a human breast tumors received an intravenous injection of iRGD or PBS before an intravenous dose of ZHS-QDs. Ag-TS was given intraperitoneally. *n* = 4 per group. **a** The mice were anesthetized and imaged with a Li-Cor Pearl imager 40 min after etching. *Arrows*, tumors. **b** In situ NIR imaging of the mice after euthanasia and necropsy performed under deep anesthesia. **c** NIR images of collected tissues. **d** NIR signal per unit area in collected tissues (*left panel*) and T/Li ratio (*right panel*). B, brain; H, heart; Li, liver; S, spleen; Lu, lung; K, kidney; T, tumor. Statistics, two-way analysis of variance (*left panel*) or Student’s *t*-test (*right panel*); *error bars*, SEM; ns, not significant; *P < 0.05; ***P < 0.001. **e** Confocal micrographs of cultured MCF10CA1a cells treated with ZHS-QDs with or without free iRGD followed by etching with Ag-TS. Note that the QDs were internalized into the cells in the presence of iRGD, and that only the extracellular QDs were etched. Blue, Hoechst 33342; green, ZHS-QDs; *scale bars*, 50 μm. *Insets* show a magnified view of the *boxed areas*. **f** Epifluorescence micrographs of cultured PC-3 human prostate cancer cells treated with cell-penetrating QDs followed by etching with Ag-TS. PC-3 cells were incubated with CdSe/ZnS QDs coated with a cell-penetrating peptide KCDGRPARPAR. Only speckled peri-nuclear signals remain after etching. *Scale bars*, 50 µm. **g** The tumors shown in **c** were processed for immunofluorescence staining and subjected to confocal microscopy. *Blue*, DAPI; *red*, CD31 or α-SMA; *green*, ZHS-QDs; *scale bars*, 50 μm. The *boxed area* is magnified. Note that the QD signals are found in the perinuclear area of the cells

The quality of breast tumor images was further assessed at 40 min post-etching. In vivo live animal imaging (Fig. 4a)*, in situ* imaging after necropsy (Fig. 4b), and *ex vivo* imaging of resected tissues (Fig. 4c) all showed tumor-specific signals in the iRGD group, while minimal fluorescence was found in the non-iRGD group. Signal intensity in the resected tumors was fivefold higher in the iRGD group than the control (Fig. 4d). T/Li ratio, a hallmark for tumor specificity, was 4.9 in the iRGD group, which is 17.5-fold higher than the average value (0.28) of previously reported inorganic nanoprobes in general, and 70-fold higher than the average value (0.07) of those with a size similar to the ZHS-QDs^3, 5^ (Fig. 4d). The T/Li ratio in the non-iRGD group was 0.98, suggesting near-equal QD intensity in the tumor and the liver.

### Effects of etching on extracellular and endocytosed QDs

These results indicate successful tumor-specific extravascular delivery of ZHS-QDs by iRGD, and effective intravascular etching by Ag-TS. However, given that Ag-TS can also extravasate to some extent through leaky tumor blood vessels (Fig. 3b)^29^, we hypothesized that the QDs in the extravascular tumor tissue were internalized in cells and that the etching was limited to the extracellular space. In a cellular context, neuropilin-binding CendR peptides such as iRGD activate an endocytic pathway similar to macropinocytosis, which helps bystander compounds to enter cells^22^. On the other hand, Ag-TS has an overall negative charge and hardly diffuses across cell membranes, making this hypothesis a valid one. In in vitro cell culture studies, iRGD allowed co-applied ZHS-QDs to enter MCF10CA1a cells (Fig. 4e). Addition of Ag-TS to the culture media led to etching of membrane-bound QDs leaving internalized QDs intact indicating that the etching was limited to the extracellular space. Similar results were obtained when Ag-TS was applied to PC-3 human prostate cancer cells pre-cultured in the presence of CdSe/ZnS-QDs coated with RPARPAR, a neuropilin-1-binding CendR peptide, with an N-terminal KCDG peptide linker (Fig. 4f)^30^. Confocal microscopy of MCF10CA1a tumor sections collected from mice, which received iRGD, ZHS-QDs, and Ag-TS, showed widespread ZHS-QDs in the extravascular tumor space (Fig. 4g). The majority of the QDs were internalized into tumor cells and also stromal cells expressing α-smooth muscle actin (α-SMA), a marker for carcinoma-associated fibroblasts (CAFs). Only minor QD signals were noted in control organs (Supplementary Fig. 4a). These results support the hypothesis that the QDs were delivered into cells in the extravascular tumor tissue, which made them resistant to subsequent etching.

### In vivo imaging of pancreatic cancer with ZHS-QDs

**Fig. 5:**
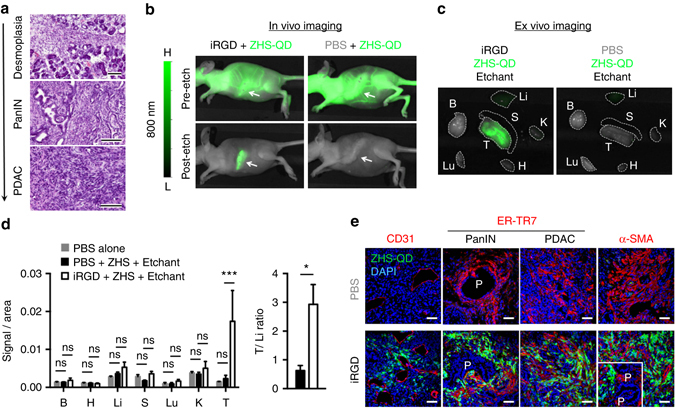
Desmoplastic PDAC imaging with etchable ZHS-QDs. **a** H&E staining of orthotopic KRAS-Ink tumor tissue collected from mice. A mixed histology ranging from pancreas tissue with severe desmoplasia, high-grade PanIN, to full-blown PDAC is observed. *n* = 3, *scale bars*, 100 μm. **b**–**e** Mice bearing orthotopic KRAS-Ink PDAC tumors received intravenous injections of iRGD or PBS 25 min before ZHS-QD injection. Ag-TS was intravenously injected 30 min after the QD injection. *n* = 3 per group. NIR images of anesthetized mice **b** and collected tissues **c**. *Arrows*, tumors; *dotted lines*, tissues. B, brain; H, heart; Li, liver; S, spleen; Lu, lung; K, kidney; T, tumor. NIR signal per unit area in collected tissues (*left panel*) and T/Li ratio (*right panel*) **d**. Statistics, two-way analysis of variance (*left panel*) or Student’s *t*-test (*right panel*); *error bars*, SEM; ns, not significant; *P < 0.05; ***P < 0.001. Confocal micrographs of tumor sections **e**. *Blue*, DAPI; *red*, CD31, ER-TR7 or α-SMA; *green*, ZHS-QDs; *scale bars*, 50 μm; P stands for PanIN. *Inset*, an area with PanINs

The finding that the iRGD/ZHS-QD system targeted CAFs prompted us to test the utility of the system for imaging desmoplastic pancreatic ductal adenocarcinoma (PDAC). We prepared mice bearing orthotopic tumors created with KRAS-Ink PDAC cells established from transgenic *p48-CRE/LSL-KrasG12D/INK4a* ^*flox*^ mice^31, 32^. The tumors develop a mixture of high-grade pancreatic intraepithelial neoplasia (PanIN) and desmoplastic PDAC^32^ (Fig. 5a). Injection of ZHS-QDs and Ag-TS resulted in minimal signals (Fig. 5b). Adding iRGD pre-injection led to a strong focal signal in the left flank where the tumor was located. Ex vivo imaging confirmed a strong tumor-specific signal (Fig. 5c). The T/Li ratio was 2.9, ~ 10 times higher than previously reported values of general inorganic nanoprobes^3^ (Fig. 5d). The QDs widely distributed in the extravascular PDAC stroma with significant co-localization with CAFs in the iRGD group (Fig. 5e). The QDs were also found around PanINs suggesting that the method could detect premalignant lesions. Only trace QD signals were noted in non-tumor tissues (Supplementary Fig. 4a). Collectively, the etchable QD system in combination with iRGD can serve as an intravenous probe to detect tumors, especially desmoplastic tumors, with high specificity.

### Intraperitoneal delivery of ZHS-QDs to peritoneal tumors

**Fig. 6:**
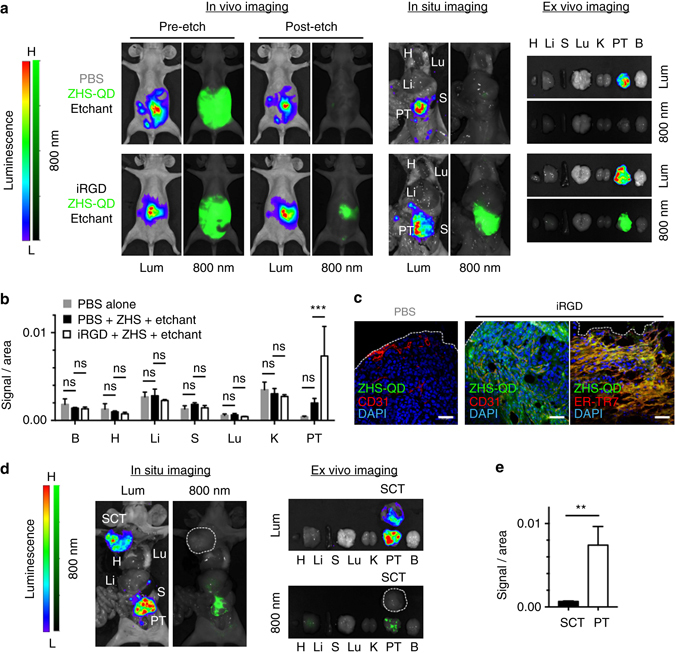
Peritoneal tumor imaging with ZHS-QDs. Mice bearing peritoneal tumors (PTs) created with MKN45P-luc luciferase-positive human gastric cancer cells received an intraperitoneal co-injection of iRGD or PBS with ZHS-QDs. Intraperitoneal etchant (1x Ag-TS) was given 90 min later. *n* = 3 per group. Some mice carried asubcutaneous MKN45P-luc tumor (SCT) in addition to the PTs. **a**, **d** Whole body in vivo and post-necropsy in situ imaging of the tumor mice, and ex vivo imaging of resected tissues. Lum, luminescence; 800 nm, NIR. The *white dotted line* in **d** marks a SCT in a mouse that received iRGD, ZHS-QDs, and Ag-TS. **b**, **e** Fluorescent signal per unit area in collected tissues **b** and in SCT vs. PT in the iRGD group **e**. **c** Confocal micrographs of PTs. *Blue*, DAPI; *red*, CD31 or ER-TR7; *green*, ZHS-QDs; *scale bars*, 50 μm. The *white dotted lines* indicate PT surface. B, brain; H, heart; Li, liver; S, spleen; Lu, lung; K, kidney. Statistics, two-way analysis of variance **b** or Student’s *t*-test **e**; *error bars*, SEM; ns, not significant; **P < 0.01; ***P < 0.001

Intraperitoneally administered iRGD facilitates penetration of co-administered compounds into peritoneal tumors in a tumor-specific manner^33^. Accordingly, the ZHS-QDs in combination with iRGD were tested for the ability to probe peritoneal tumors created with luciferase-positive MKN45P-luc human gastric cancer cells in mice^33^. Intraperitoneal co-injection of iRGD and ZHS-QDs led to strong fluorescence localized in the abdomen of the peritoneal tumor mice (Fig. 6a). No obvious extraperitoneal signal was noted. Subsequent intraperitoneal injection of Ag-TS abolished most of the fluorescence within 5 min leaving a focal signal, which co-localized with luminescent peritoneal tumors. The peritoneal tumor-specific probing was confirmed by *in situ* imaging of the mice (Fig. 6a) and *ex vivo* imaging of resected tissues (Fig. 6a). Signal intensity in the resected peritoneal tumors was threefold higher in the iRGD group than those in the control group (Fig. 6b). Confocal microscopy showed strong QD signals in peritoneal tumors especially in stroma-rich areas (Fig. 6c). Almost no QDs were found in non-tumor tissues (Supplementary Fig. 4b). Intraperitoneal injection of ZHS-QDs and Ag-TS without iRGD did not lead to tissue probing.

The lack of systemic NIR signals in the peritoneal tumor mice that received iRGD and ZHS-QDs suggested that the QDs entered the tumors predominantly through local penetration and not through the circulation. Indeed, intraperitoneal co-administration of iRGD promotes local entry of dextran and small-molecule drugs into peritoneal tumors in a circulation-independent manner^33^. However, iRGD-coated nanoparticles have been shown to enter the circulation from the abdominal cavity, and target peritoneal tumors through a combination of both circulation-dependent and -independent pathways^34, 35^. To separate the two pathways in the peritoneal tumor targeting by iRGD/ZHS-QDs, we tested the efficacy of extraperitoneal tumor targeting by intraperitoneally administered iRGD/ZHS-QDs in mice bearing a subcutaneous MKN45P-luc tumor in addition to the peritoneal tumors. Intraperitoneal injections of iRGD and ZHS-QDs followed by Ag-TS led to strong NIR signals in the peritoneal tumors, while the subcutaneous tumor remained macroscopically unprobed (Fig. 6d). Quantification of fluorescence intensity in the resected subcutaneous and peritoneal tumors further confirmed the results (Fig. 6e). These results strongly suggest that the ZHS-QDs minimally entered the circulation, and if any, circulating ZHS-QDs had minor effects on peritoneal tumor targeting after intraperitoneal co-injection of iRGD and ZHS-QDs. Thus, the peritoneal tumor-specific probing was likely a result of local QD entry into the tumors facilitated by iRGD. These results indicate that local delivery of etchable QDs into peritoneal tumors followed by etching of excess QDs in the abdominal cavity allows peritoneal tumor-specific imaging. Collectively, the etchable QD system in combination with iRGD can be a powerful diagnostic platform and an aid in debulking surgeries for peritoneal carcinomatosis^33, 36^, in addition to serving as an intravenous imaging probe for various types of tumors.

### Synthesis and characterization of Hg-free etchable QDs

**Fig. 7:**
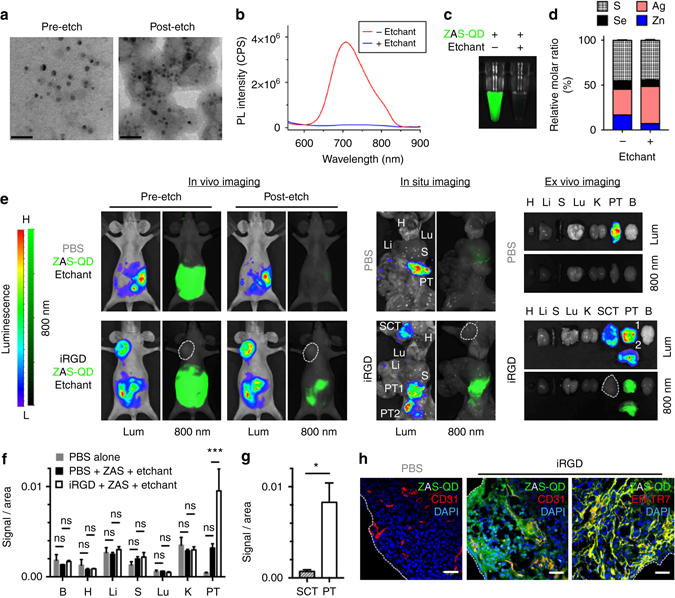
Peritoneal tumor imaging with Hg-free etchable ZAS-QDs. **a**–**c** ZAS-QDs characterized by TEM **a**, PL spectra (excitation: 460 nm) **b** and NIR imaging with a Li-Cor Pearl imager (green, ZAS-QDs) **c** before and after Ag-TS treatment. *Scale bars*, 50 nm. **d** EDS analysis of ZAS-QDs before and after Ag-TS treatment showed relatively constant S and Se and increased Ag after etching. See Supplemental Table 1 for complete data set. *n* = 3 per group. **e**–**h** Mice bearing peritoneal tumors (PTs) created with MKN45P-luc luciferase-positive human gastric cancer cells received intraperitoneal co-injection of iRGD or PBS with ZAS-QDs followed by Ag-TS. *n* = 3 per group. In **e**, live in vivo and post-necropsy in situ images of the tumor mice, and ex vivo images of resected tissues are shown. Lum, luminescence; 800 nm, NIR. *The white dotted line* marks a subcutaneous tumor (SCT).NIR signal per unit area in collected tissues **f** and in SCT vs. PT in the iRGD group **g**. B, brain; H, heart; Li, liver; S, spleen; Lu, lung; K, kidney. Statistics, two-way analysis of variance **f** or Student’s *t*-test **g**; *error bars*, SEM; ns, not significant; *P < 0.05; ***P < 0.001. Confocal micrographs of PTs **h**. Blue, DAPI; red, CD31 or ER-TR7; green, ZAS-QDs; *scale bars*, 50 μm; *white dotted lines* in **h**, PT surface

Given the success in highly tumor-specific imaging using the etchable ZHS-QD system, we set out to improve the system by synthesizing Hg-free PEGylated QDs to avoid any potential Hg exposure in vivo. The QDs, ZAS-QDs, consisted of Zn, Ag, Se and S, as confirmed by EDS analysis (Supplementary Table 1). The core diameter of the ZAS-QDs was 6.8 ± 4.3 nm based on TEM measurements (Fig. 7a and Supplementary Fig. 5a). The PL emission extended beyond 800 nm with a peak at 708 nm, which made the QDs applicable to NIR imaging (Fig. 7b, c, and Supplementary Fig. 5b). The QY was 2%.

Ag-TS abolished the PL in seconds in vitro, and slightly reduced the core size to 5.2 ± 1.8 nm (Fig. 7a–c, Supplementary Table 2 and Supplementary Fig. 5a). Elemental analysis showed that the Ag to Zn ratio significantly increased upon Ag-TS treatment without major changes in the anion ratio indicating that cation exchange took place (Fig. 7d and Supplementary Fig. 5c). Nearly 90% of C and O remained after etching indicating that the PEG layer largely remained associated with the QD surface (Supplementary Table 1). Crystallographic analysis with XRD of the QDs showed a peak shift towards a higher angle with the emergence of peaks for Ag_2_S and/or Ag_2_Se after Ag-TS treatment, supporting the mechanism of quenching by Ag ion exchange in the QD core (Supplementary Fig. 5d).

### In vivo imaging of peritoneal tumors with ZAS-QDs

The ZAS-QDs were tested in mice bearing MKN45P-luc peritoneal tumors for in vivo imaging. Intraperitoneally injected ZAS-QDs were strongly detected in live animals with a Li-Cor Pearl imager under an 800 nm channel (Fig. 7e). The NIR signal was limited to the abdominal cavity suggesting minimal entry of ZAS-QDs into systemic circulation. Subsequent intraperitoneal injection of Ag-TS eliminated the signal within 5 min. Almost no fluorescence was noted in peritoneal tumors suggesting that there was minimal tumor entry of the QDs. When iRGD was given together with the ZAS-QDs and subsequent intraperitoneal etching was performed, strong fluorescent signals co-localizing with luminescent peritoneal tumors were found. In situ imaging of abdominal and thoracic organs and ex vivo imaging of resected tissues (Fig. 7e) confirmed the peritoneal tumor specificity of the NIR signals. Almost no NIR signal was noted in extraperitoneal subcutaneous tumors suggesting that the ZAS-QDs entered the peritoneal tumors through a circulation-independent fashion (Fig. 7e–g). Consistent with these findings, confocal microscopy showed strong QD signals on peritoneal tumor surfaces with decreasing signals extending into the tumor interior (Fig. 7h). The majority of the QD signals were associated with nucleated cells. Importantly, there was significant co-localization with a reticular fibroblast marker suggesting that the stroma, mainly consisting of fibroblasts, served as a conduit for QD penetration into the tumors. In subcutaneous tumors, some QD-positive blood vessels were present suggesting that the QDs entered the circulation to some extent at microscopic levels (Supplementary Fig. 4b). QDs were not evident in non-tumor tissues. Thus, the Hg-free ZAS-QDs have etchable properties similar to ZHS-QDs, and provide highly tumor-specific signals when used in combination with iRGD and the Ag-TS etchant, demonstrating their capacity in tumor-specific in vivo imaging.

## Discussion

**Fig. 8:**
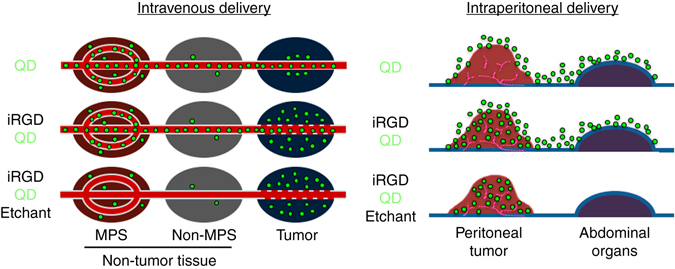
Tumor-specific imaging with etchable QDs. (Intravenous delivery) Schematic of tumor-specific imaging with tumor-penetrating etchable QDs delivered through the circulation. The schematic depicts an early post-etching time point. *Top panel*: QDs (*green particles*), when injected alone, eventually accumulate mainly in the MPS by passing through the rich sinusoids. Some QDs may enter the extravascular tumor tissue because of the EPR effect. *Middle panel*: Pre-injection of iRGD facilitates extravasation and cell internalization of QDs in a tumor-specific manner. The QDs also eventually accumulate in the MPS. *Bottom panel*: Etching performed soon after the iRGD-mediated QD delivery into extravascular tumor cells causes quenching of intravascular and extracellular QDs leading to highly tumor-specific signals. (Intraperitoneal delivery) Schematic of peritoneal tumor imaging with intraperitoneally delivered etchable QDs. *Top panel*: intraperitoneal QDs attach to the tumor and peritoneal surfaces. *Middle panel*: iRGD facilitates local penetration of QDs specifically into peritoneal tumors. *Bottom panel*: Intraperitoneal etching differentially quenches the QDs that did not enter the tumors without affecting the intratumoral QDs, leading to highly tumor-specific signals

This study introduces an important concept in imaging whereby probe brightness is controlled in vivo with a biocompatible chemical reaction. We have achieved exceptionally tumor-specific imaging by delivering etchable QDs specifically into tumor tissue through intravenous and intraperitoneal routes followed by preferential etching of excess counterparts in non-tumor tissues (Fig. 8). The differential etching is enabled by the contrast of QD dynamics between the rapid and active intratumoral spreading facilitated by iRGD, and the slow and passive accumulation into normal tissues^5, 37^. In particular, performing the etching procedure soon after achieving tumor-specific targeting of PEGylated QDs can minimize their non-specific entry into the MPS (e.g., liver and spleen). The lack of etching of intratumoral QDs can be attributed to a combination of endocytosis, which protects QDs from a membrane-impermeable etchant, and the transient time window for iRGD to deliver QDs into tumor tissue and cells before subsequent etching is performed^21^.Compared to our previous Ag nanoparticle-based system that releases a dye upon etching^38^, it is noteworthy here that low background was achieved by directly modifying the chemical composition of the emitting species—a concept that may be extended to other fluorophores and imaging modalities. Although a metal-free etchable platform with a dissolvable core is ultimately desired for human use, this proof-of-concept study lays the groundwork for next-generation nanoprobes with extremely high tumor-specificity.

## Methods

### Materials

Zinc acetate dihydrate [Zn(OAc)_2_· 2H_2_O,99.9%], mercury(II) perchlorate hydrate [Hg(ClO_4_)_2_· xH_2_O, 99.998%], poly(ethylene glycol) methyl ether thiol (CH_3_O-PEG2000-SH, Mw 2000), silver nitrate (AgNO_3_, ≥ 99.0%), sodium chloride (NaCl), potassium chloride (KCl,  ≥ 99.0%), manganese(II) chloride tetrahydrate (MnCl_2_· 4H_2_O,_ ≥ _99%), magnesium chloride (MgCl_2_, ≥ 98%), iron(III) chloride hexahydrate (FeCl_3_· 6H_2_O, 97%), potassium hexacyanoferrate(III) [K_3_Fe(CN)_6_, ≥ 99.0%], potassium hexacyanoferrate(II) trihydrate [K_4_Fe(CN)_6_·3H_2_O, 98.5–102.0%], sodium borohydride (NaBH_4_, 99%),sodium thiosulfate pentahydrate (Na_2_S_2_O_3_· 5H_2_O, ≥ 99.5%), copper(II) sulfate pentahydrate (CuSO_4_· 5H_2_O, ≥ 98.0%), D-penicillamine (98–101%) and sodium 2,3-dimercaptopropanesulfonate monohydrate (DMPS, ~ 95%) were purchased from Sigma-Aldrich (St. Louis, MO). Thiol PEG amine (HS-PEG2000-NH_2_, Mw2000) HCl salt was purchased from JenKem Technology USA (Plano, TX). Amphiphilic ligand HS-(CH_2_)_11_-(OCH_2_CH_2_)_6_-NH_2_ was purchased from ProChimia (Gdansk, Poland). Selenium powder (−325 mesh, 99.5%) was purchased from Alfa Aesar (Ward Hill, MA). Ammonium bicarbonate (NH_4_HCO_3_) and 3-mercaptopropionic acid (≥99.0%) were from Fisher Scientific (Pittsburgh, PA). Sodium hydroxide (NaOH) pellets were bought from Amresco (Solon, OH). iRGD and CRGDC peptides were synthesized in house^21^. H_2_O was purified using a Milli-Q (EMD Millipore) system.

### Cell lines and mouse models

MCF10CA1a human breast cancer cells^39^, KRAS-Ink mouse PDAC cells^31^, PC-3 human prostate cancer cells^30^, and MKN45P-luc luciferase-positive human gastric cancer cells^33^were cultured in Dulbecco’s modified Eagle’s medium supplemented with 10% fetal bovine serum, 100 U ml^−1^ penicillin and 100 µg ml^−1^ streptomycin. The human cell lines were authenticated by the DNA Analysis Core Facility at the Sanford Burnham Prebys Medical Discovery Institute (La Jolla, CA) and the KRAS-Ink cell line was authenticated by DDC Medical (Fairfield, OH). All the cells were tested negative for mycoplasma contamination. Female BALB/c and athymic nude mice were both purchased from Harlan Laboratories (Indianapolis, IN). Orthotopic breast tumors were generated by injecting 2 × 10^6^ MCF10CA1a cells into the mammary fat pad^39^, and orthotopic PDAC tumors were generated by injecting 2 × 10^6^KRAS-Ink cells into the pancreas^20^ of female nude mice at 10 weeks of age. Peritoneal tumor mice were generated by injecting 10^7^ MKN45P-luc cells into the peritoneal cavity of female nude mice at 10 weeks of age^33^. All animal procedures were performed according to protocols approved by the Institutional Animal Care and Use Committee at Sanford Burnham Prebys Medical Discovery Institute.

### Synthesis and characterization of QDs

ZHS-QDs were synthesized based on a previously reported method with modifications^40^. Specifically, CH_3_O-PEG2000-SH powder (360 mg) was added into NH_4_HCO_3_ solution (2.4 ml, 0.2 M, pH = 12.4 adjusted with 5 M NaOH) and completely dissolved after short sonication. Zn(OAc)_2_· 2H_2_O (900 μl, 100 mM) and Hg(ClO_4_)_2_· xH_2_O (270 μL, 100 mM) were then added into the solution and quickly mixed, followed by immediate addition of freshly prepared sodium hydrogen selenide (NaHSe, 180 μl, 250 mM)^40^. Adding NaHSe caused the reaction solution to turn dark instantly, indicating QD formation. After mixing in the dark at room temperature (RT) for 1 h, the solution was moved to 4 °C for passivation for 48 h. After centrifugation (11,000 × *g*, 10 min, 4 °C), the supernatant containing dispersed QDs was collected, neutralized with sodium acetate-acetic acid buffer (pH 5.0, 1 M), and purified six times in PBS by ultrafiltration with Ultra-4 10 kDa centrifugal filters (EMD Millipore, Darmstadt, Germany). After another centrifugation (11,000 × *g*, 10 min, 4 °C), the supernatant was filtered with a 0.22 μm syringe filter, and kept in the dark at 4 °C until use.

Positively charged ZHS-QDs (ZHS-NH_2_) were prepared via the same procedure as above except that CH_3_O-PEG2000-SH was replaced with HS-PEG2000-NH_2_. Mn/ZnS QDs were synthesized as previously reported^41^. CdSe/ZnS core/shell QDs were purchased from eBioscience (San Diego, CA). InP/ZnS QDs and CIS/ZnS QDs, both coated with 3-mercaptopropionic acid, were bought from NNCrystal US Corporation (Fayetteville, AR). All the QDs were water-soluble. CdSe/ZnS QDs coated with cell-penetrating KCDGRPARPAR peptides (LifeTein, Somerset, NJ) were prepared according to the manufacturer’s instructions associated with the eFluor 605NC sulfhydryl-reactive conjugation kit (eBioscience). CIS/ZnS-QDs coated with HS-(CH_2_)_11_-(OCH_2_CH_2_)_6_-NH_2_ were prepared as described earlier^42^.

ZAS-QDs were synthesized by first adding 3-mercaptopropionic acid (10 µl) and Zn(OAc)_2_· 2H_2_O (75 μl, 100 mM) to NH_4_HCO_3_ solution (0.2 ml, 0.2 M, pH = 12.8 adjusted with 5 M NaOH). After vortexing for 30 s, CH_3_O-PEG2000-SH (45 mg) and DMPS (5.4 mg) in H_2_O (0.2 ml, 4 °C) were added to the solution, and the solution was vortexed again for 30 s. Finally, NaHSe (15 μl, 250 mM) was added, followed by immediate addition of AgNO_3_ (75 μl, 200 mM) and vortexing for 30 s. After mixing in the dark at RT for 1 h, the solution was moved to 4 °C for overnight passivation. The solution was centrifuged (11,000  × *g*, 10 min, 4 °C), and the supernatant was washed first in H_2_O, then in PBS, by ultrafiltration with Amicon Ultra-4 centrifugal filters (NMWL 10 KDa) until the pH of the filtrate neutralized. After another centrifugation (11,000 × *g*, 10 min, 4 °C), the supernatant was passed through a 0.22 μm syringe filter, and kept in the dark at 4 °C until use. The concentrations of all ZHS-QDs and ZAS-QDs samples described in this study are expressed as Zn^2+^ concentration unless otherwise stated.

TEM was performed with a JEM-1200EX II electron microscope (JEOL, Tokyo, Japan) operating at 80 kV. Absorbance spectra were measured with a DU800 spectrophotometer (Beckman Coulter, CA), and PL spectra were measured with a FluoroMax-4 spectrofluorometer (Horiba, Kyoto, Japan). QYs for ZHS- and ZAS-QDs were determined by using Rhodamine 6 G as standard (QY = 95% in ethanol). The QY values for CdSe/ZnS-, InP/ZnS- and CIS/ZnS-QDs were acquired from the commercial source. EDS spectra were acquired using aXL30-SFEG UHR scanning electron microscope (FEI, Hillsboro, OR) equipped with a Silicon Drift Detector iXRFEDS analyzer (E2V, Chelmsford, Essex, UK), operating at 10 kV. XRD measurementswere performed on a MiniFlex II X-ray diffractometer (Rigaku, Tokyo, Japan) with Cu-Ka radiation (*λ* = 0.15418 nm) at 30 kV and 15 mA. ICP-OES measurement was performed with an Optima 3000 DV ICP-OES spectrometer (Perkin-Elmer, Waltham, MA). DLS was carried out with Malvern Zetasizer Nano ZS90 (Worcestershire, UK) to determine the hydrodynamic size of the QDs.

### Synthesis of etchants and in vitro etching studies

Ag-TS was synthesized as follows. AgNO_3_(0.08 mmol) was added into PBS (500 μl) and mixed for 30 s forming a precipitate. Na_2_S_2_O_3_ in H_2_O (0.2 M, 1 ml) was then added to the solution and mixed for 5 min to allow the precipitate to dissolve. After filtration with a 0.22 μm syringe filter, a clear colorless solution was obtained (0.05 M Ag-TS stock solution, designated as ‘10×’). The solution was diluted 5 or 10 times with PBS to obtain a 2× or 1x Ag-TS solution, which was used on the same day for in vivo etching studies.

To study the principle of etching, ZHS-QDs or ZAS-QDs in PBS (7.5 mM, 350 μl) were mixed with either PBS (100 μl) or 10x Ag-TS etchant (100 μl) for 1 h at RT, followed by ultrafiltration in H_2_O with Amicon Ultra-0.5 centrifugal filters (NMWL 10 kDa). The filtrates and concentrates were collected separately for ICP-OES measurement^42^. The collected concentrates were also subjected to TEM observation and PL spectra measurement.

Aqueous solutions of various chemicals were prepared to examine their etching capacity in vitro. The concentration was 1 mM for all the chemicals except for Na_2_S_2_O_3_ (Na-TS), which was 2.5 mM, and Ag-TS and Cu-TS, which were prepared by mixing AgNO_3_ (2 mM) or CuSO_4_ (2 mM) with an equal volume of Na-TS (5 mM). Each chemical (400 µl) was mixed with ZHS-QDs (400 µl, 0.5 mM of Zn^2+^) at RT for 1 min. Immediately, the mixtures were imaged with a Li-Cor Pearl imager under the 800 nm channel, and the PL spectra of the mixtures were also immediately measured with an excitation wavelength of 450 nm. Emission peak intensity was used to calculate relative PL intensity. A mixture of ZHS-QD solution (400 µl, 0.5 mM) and H_2_O (400 µl) was used as a positive control, the relative PL intensity of which was considered as 100%.

To study time-dependent quenching kinetics of ZHS-QDs, the QDs in PBS (1 ml) with Zn concentration of 5 µM was added into a cuvette. Fluorescence was collected at regular time intervals, showing a steady fluorescence signal. Ag-TS (2 mM of Ag) was then quickly mixed in the sample in the cuvette to a final Ag concentration of 5 µM. A Horiba FluoroMax-4 spectrofluorometer was used at 450 nm excitation (2 nm slit width) and 650 nm emission (5 nm slit width) with integration time of 0.5 s per point. To correct for background signal, the intensity of PBS using these settings was measured and subtracted from all data points. To study stoichiometric quenching kinetics, equation (2) was used for different molar ratios of Ag to Zn:

2 $$\begin{array}{*{20}{l}}{\rm{ZHS}}\,{\rm{quenched}}\left( {\rm{\% }} \right) = 100 \times \left( {1 - \frac{{{\rm{PL}}\,{\rm{intensity}}\,{\rm{of}}\,{\rm{ZHS}} - {\rm{QD}}\,{\rm{with}}\,{\rm{Ag}} {\hbox{-}} {\rm{TS}} \,-\, {\rm{background}}}}{{{\rm{PL}}\,{\rm{intensity}}\,{\rm{of}}\,{\rm{ZHS}} - {\rm{QD}}\,{\rm{without}}\,{\rm{Ag}} {\hbox{-}} {\rm{TS}} \,-\, {\rm{background}}}}} \right) \hfill \end{array}$$

All the experiments were performed at room temperature.

To study the etchability of different QDs, aqueous solutions (400 µl per solution) of Mn/ZnS (Zn concentration: 10 mM), InP/ZnS (0.25 mg ml^−1^) and 3-mercaptopropionic acid-coated and HS-(CH_2_)_11_-(OCH_2_CH_2_)_6_-NH_2_-coated CIS/ZnS (0.5 mg ml^−1^) QDs were added with an equal volume of 1x Ag-TS or H_2_O, while CdSe/ZnS (QD concentration: 3.2 nM) in H_2_O (400 µl) was added with an equal volume of 10× Ag-TS or H_2_O. After mixing at RT for 5 min, the mixtures were subjected to PL imaging and PL spectra measurement. PL imaging was performed with an UV/White light transilluminator LMW-20 (UVP, Upland, CA) for Mn/ZnS QDs, an Illuma tool Bright Light System LT-9900 (Lightools Research, Encinitas, CA) for CdSe/ZnS and InP/ZnS QDs and HS-(CH_2_)_11_-(OCH_2_CH_2_)_6_-NH_2_-coated CIS/ZnS QDs, and a Li-Cor Pearl imager under 700 nm channel for 3-mercaptopropionic acid-coated CIS/ZnS QDs.

### In vitro QD uptake by human tumor cells

PC-3 cells, which express neuropilin-1, a cell surface receptor for the KCDGRPARPAR peptide^30^, were incubated in 96-well plates with KCDGRPARPAR-coated CdSe/ZnS QDs (QD concentration: 25 nM) for 90 min at 37 °C. The cells were subjected to epifluorescence imaging with a Leica DMIRE2 microscope (Leica, Wetzlar, Germany) before and after the addition of 1x Ag-TS (1 µl) to each well containing 100 µl of culture media. MCF10CA1a cells were incubated with or without iRGD peptide (final concentration: 50 µM) in culture media in chambered coverglass (Nunc Lab-Tek II, Rochester, NY) for 30 min at 37 °C, and ZHS-QDs were added to each chamber (final concentration: 1 mM Zn^2+^). After incubation for 2.5 h at 37 °C, the cells were washed once with PBS, and cultured in fresh culture media containing Hoechst 33342 (10 µg ml^−1^ in 400 µl, Molecular Probes, Eugene, OR) for 10 min at 37 °C. Etching was performed by adding 1x Ag-TS (100 µl) to each chamber and incubating the cells for 1 min at RT. The cells were imaged with a Zeiss LSM 710 NLO confocal microscope (Carl Zeiss, Oberkochen, Germany) before and after etching.

### Biodistribution, clearance, and in vivo toxicity

BALB/c female mice10–12 weeks of age were intravenously injected with 100 μL of PBS or ZHS-QDs in PBS (18 nmol Zn per g body weight) followed 30 min later with intraperitoneal injection of 300 μL of PBS or 1x Ag-TS. For biodistribution and clearance studies, the mice were sacrificed under deep anesthesia 1 h, 24 h, and 10 days after Ag-TS injection. Major tissues, serum, feces and urine samples were collected for quantification of mercury with ICP-OES^42^. To examine in vivo toxicity, blood was collected from the retro-orbital plexus of each mouse under deep anesthesia into a lithium-heparin 1.3 ml micro tube (Sarstedt, Nümbrecht, Germany) after 24 h and 1 week, and plasma was isolated by centrifugation. The plasma (100 μl) was pipetted into a Comprehensive Diagnostic Profile Rotor (Product Part No. 500-1038, Abaxis, Union City, CA) and subjected to biochemical toxicity assays using an AbaxisVetScan VS2 chemistry analyzer. The mice were then sacrificed under deep anesthesia, and tissues were collected and processed for paraffin embedding. Sectioned paraffin blocks were stained with hematoxylin and eosin (H&E), and whole slide scanning was performed with a Leica SCN 400 slide scanner.

### Imaging of normal mice

Female nude mice without tumors were anesthetized with isoflurane and imaged with an 800 nm channel with a Pearl Impulse small animal imaging system (Li-Cor, Lincoln, NE) before any injection. Then, the mice were intravenously injected with ZHS-QDs at a dose of 18 nmol Zn per g body-weight per injection. Some mice received an intravenous dose of PBS (100 μl) or iRGD (100 μl, 2.5 mM in PBS) 25 min before the QD injection. Subsequent etching was performed at time points indicated in each Figure by either anintravenous injection of 1x Ag-TS (100 μl), intraperitoneal injection of 1× Ag-TS (300 μl), or three consecutive subcutaneous injections of a mixture of D-penicillamine and CuSO_4_ at a volume of 50 μl per injection. The penicillamine/CuSO_4_solution was prepared by mixing stock solutions of D-penicillamine (40 mM) and an equal volume of CuSO_4_ (20 mM) just prior to use and without filtration. We used a higher intraperitoneal dose of Ag-TS than that of intravenous Ag-TS because intraperitoneal delivery generally leads to lower peak plasma concentrations than intravenous delivery^13^. In some cases, the mice were sacrificed under deep anesthesiafor tissue collection.

### Imaging of breast tumors and PDAC in mice

Pre-injection imaging of mice bearing an orthotopic MCF10CA1a breast tumor or KRAS-Ink PDAC was performed under anesthesia with isoflurane using a Pearl Impulse imager equipped with an 800 nm channel. The mice were intravenously injected with PBS (100 μl) or iRGD or CRGDC in PBS (100 μl, 2.5 mM), followed by an intravenous injection of ZHS-QDs in PBS (100 μl) at a dose of 18 nmol Zn per g body-weight 25 min later. Subsequent etching was performed by an intraperitoneal injection of 300 μl of 1× Ag-TS in breast tumor mice or an intravenous injection of 100 μl of 2x Ag-TS in PDAC mice. In some cases, ZHS-NH_2_QDs in PBS (100 μl) at a dose of 18 nmol Zn per g body-weight were injected instead of ZHS-QDs. The mice were anesthetized and imaged at different time points as shown in each Figure. After in vivo imaging, the mice were sacrificed under deep anesthesia. Breast tumor mice received cardiac perfusion of PBS, while PDAC mice did not. The necropsied mice (in situ imaging) and resected tissues (ex vivo imaging) were imaged with the Pearl Impulse small animal imaging system. The tissues were processed for immunofluorescence as described elsewhere^20^. In some cases, PDAC tissues were embedded in paraffin for H&E staining and subsequent histology analysis as described elsewhere^21^. Fluorescence intensity quantification was performed with Li-Cor Image Studio Lite 4.0 software to calculate CI, fluorescent signal per tissue area, and T/Li ratios.

### Imaging of peritoneal tumor in mice

Mice bearing MKN45P-luc peritoneal tumors were intraperitoneally injected with ZAS-QDs (30 nmol Zn per g body-weight) or ZHS-QDs (45 nmol Zn per g body-weight) in PBS (500 μl) with or without 450 μg of iRGD. At 70 min postinjection of QDs, the mice were intraperitoneally injected with luciferin (15 mg ml^−1^ in PBS, Biosynth International, Itasca, IL) at a dose of 0.28 mg per g body-weight. The mice were then anesthetized with isoflurane, and imaged at different time points for luminescence with a Xenogen IVIS imager (Perkin-Elmer) and for NIR with a Li-Cor Pearl Impulse imager under 800 nm channel. Some mice also had an extraperitoneal subcutaneous MKN45P-luc tumor in the presence of the peritoneal tumors. At 90 min postinjection of QDs, 1× Ag-TS (400 μl) was intraperitoneally injected. After 5 min, the mice were imaged with the Xenogen IVIS and Li-Cor Pearl Impulse imagers. The mice were then immediately sacrificed under deep anesthesia by cardiac perfusion with PBS, and the necropsied mice (in situ imaging) and resected tissues (ex vivo imaging) were imaged again. The abdominal cavity of the necropsied mice was not washed before in situ imaging. The tissues were processed for immunofluorescence as described elsewhere^33^. Fluorescence intensity quantification was performed with Li-Cor Image Studio Lite 4.0 software.

### Immunofluorescence

Tissue sections were treated with 0.25% Triton X-100 for 10 min, washed with PBS 3 times, blocked with 1% bovine serum albumin for 1 h, and incubated with a rat anti-mouse CD31 primary antibody (Catalog number: 553370, BD Biosciences, San Jose, CA), rabbit anti-mouse α-SMA antibody (Product code: ab5694, Abcam, Cambridge, MA), or a rat anti-mouse ER-TR7 antibody (Catalog number: sc-73355, Santa Cruz Biotechnology, Dallas, TX) at 4 °C overnight. Secondary antibodies were Alexa Fluor 488 goat anti-rat IgG (Catalog number: A-11006) and Alexa Fluor 488 goat anti-rabbit IgG (Catalog number: A-11034)(Thermo Fisher Scientific, Rockford, IL). After washing with PBS, sections were mounted in DAPI-containing mounting medium (Vector Laboratories, Burlingame, CA) and examined under a Zeiss LSM 710 NLO confocal microscope.

### Statistical analysis

At least three mice were analyzed per group. The mice were randomly assigned to each study group. No sample size estimates or blinding techniques were used. All statistical analyses were conducted with GraphPad Prism 7 or Excel (*P < 0.05, **P < 0.01, ***P < 0.001; ns, not significant). Unless otherwise stated, all data are expressed as mean ± standard error of the mean (SEM). Statistical analysis was performed using Student’s two-tailed *t*-test or one-way or two-way analysis of variance followed by *post hoc* analysis. A value of *P* *<* 0.05 was considered statistically significant.

### Data availability

The authors declare that the data of this study are available within the article and its Supplementary Information, or from the corresponding authors upon reasonable request.

## Electronic supplementary material

Supplementary Information
