# Supplementary material for: In vivo cation exchange in quantum dots for tumor-specific imaging
Source: Nat Commun. 2017 Aug 24;8:343. doi: 10.1038/s41467-017-00153-y (PMC5571182; doi:10.1038/s41467-017-00153-y)
Supplement: Supplementary file 1 — Supplementary Information [file 41467_2017_153_MOESM1_ESM.pdf]

SI GUIDE

File Name: Supplementary Information
Description: Supplementary Figures and Supplementary Tables.

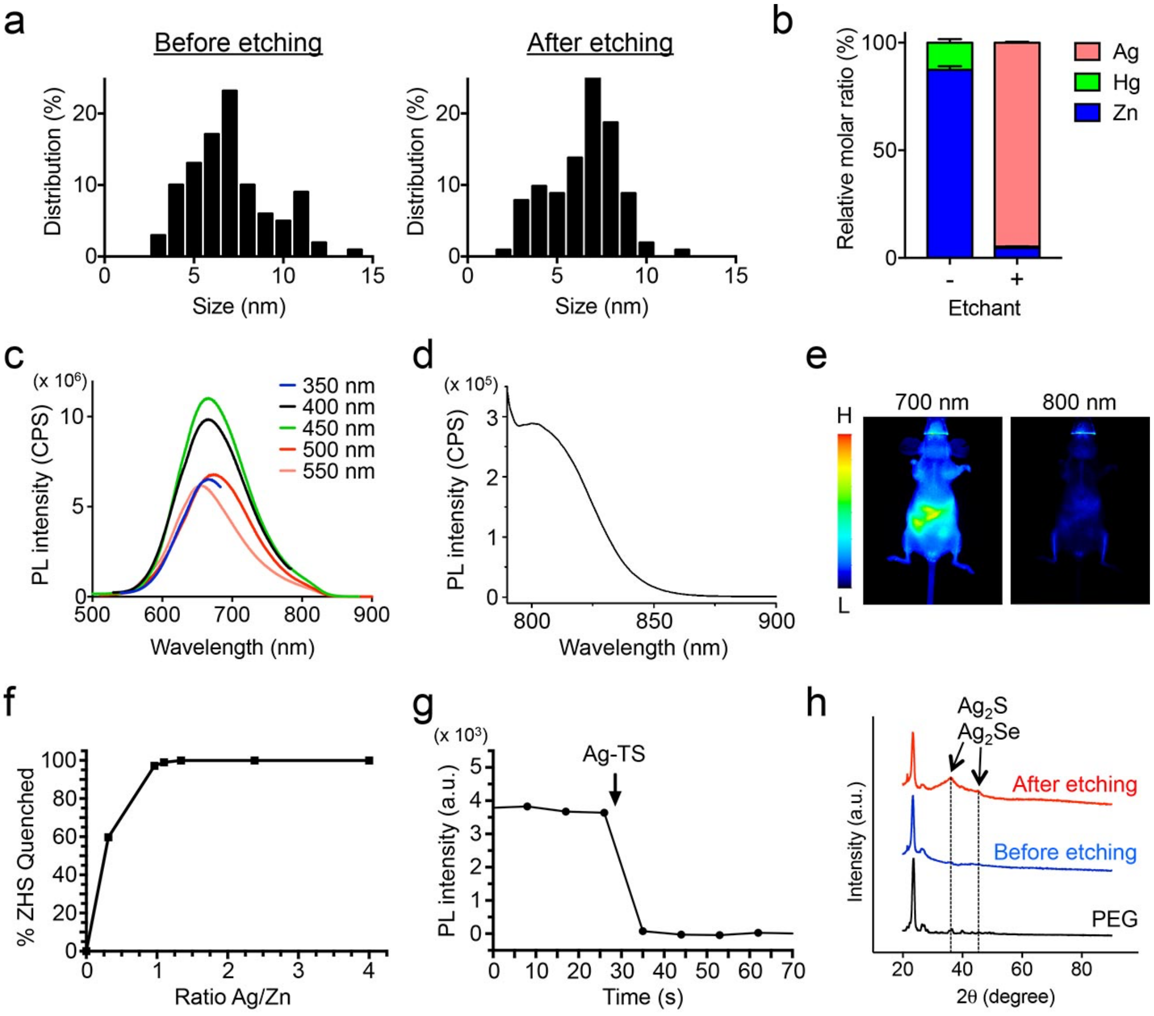

Supplementary Figure 1. Additional characteristics of ZHS-QDs. (**a, b, h**) TEM-based size distribution analyses (a), elemental analysis with ICP-OES (b), and crystal structure analysis with XRD (h) of ZHS-QDs before and after Ag-TS treatment. In (b), n = 3 per group. (**c, d**) PL spectra of ZHS-QDs at various excitation wavelengths (c) or 785 nm excitation (d). Note that in (d), emission wavelength range excluded the 785 nm region to avoid saturation artifacts on the detector by the excitation light. (**e**) A plain normal mouse imaged with a Li-Cor Pearl imager using 700 nm and 800 nm channels. Note that the 700 nm channel provides significant autofluorescence, while the 800 nm channel does not. (**f, g**) Stoichiometric (f) and time-dependent (g) quenching kinetics of ZHS-QDs with Ag-TS was measured in aqueous solution at room temperature. A Horiba FluoroMax-4 was used at 450 nm excitation (2 nm slit width) and 650 nm emission (5 nm slit width) with integration time of 0.5 s per point. In (g), ZHS-QDs were mixed with Ag-TS at an Ag to Zn molar ratio of 1:1.

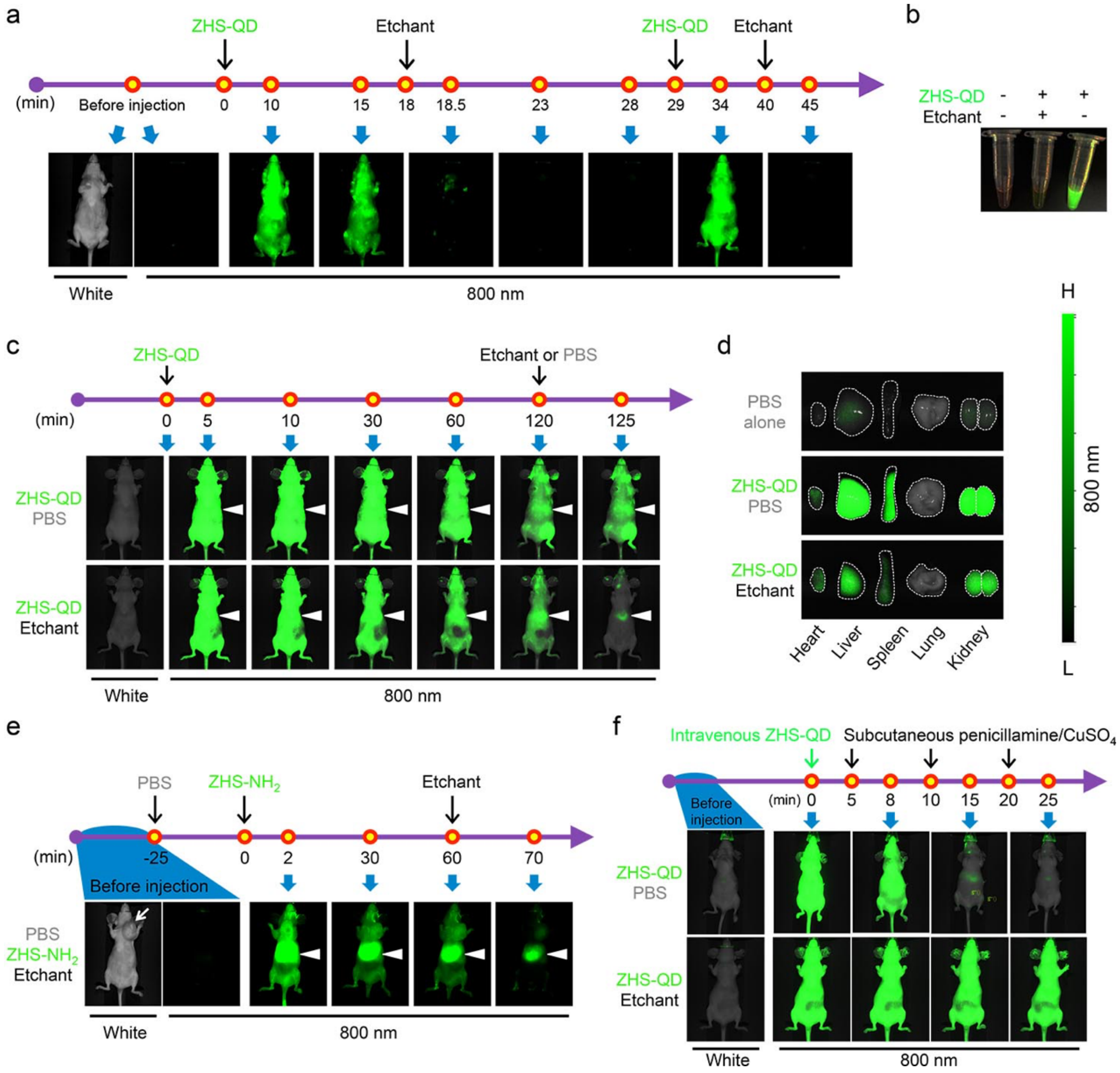

Supplementary Figure 2. *In vivo* etching of ZHS-QDs achieved with etchants injected through various routes. Nude mice received intravenous ZHS-QDs followed by PBS or an etchant (Ag-TS or penicillamine/$CuSO_4$) at indicated time points from different injection routes, and were subjected to longitudinal NIR imaging from the ventral side. n = 3 per study. (**a**) Alternating intravenous doses of ZHS-QDs and Ag-TS were given. (**b-d**) Intraperitoneal Ag-TS was given 30 min (b) or 2 h (c, d) after an intravenous dose of ZHS-QDs. In (b), serum samples collected 1 h after etching were imaged. In (d), major organs were collected at the last time point in (c), and imaged for NIR. Dotted lines, tissues; arrowheads, liver. (**e**) Intraperitoneal Ag-TS was given 60 min after an intravenous dose of ZHS-NH_2 QDs (ZHS-QDs have positive surface charge and show rapid liver accumulation). Note the bright liver signal after etching (arrowhead). The partial decrease in the signal can be attributed to quenching of intravascular QDs in the liver sinusoids. An orthotopic MCF10CA1a breast tumor is nearly undetectable (arrow). (**f**) Three consecutive subcutaneous doses of a mixture of penicillamine and $CuSO_4$ at a molar ratio of 2:1 were given at the indicated time points. Note the near complete etching achieved within approximately 20 min from the first etchant injection.

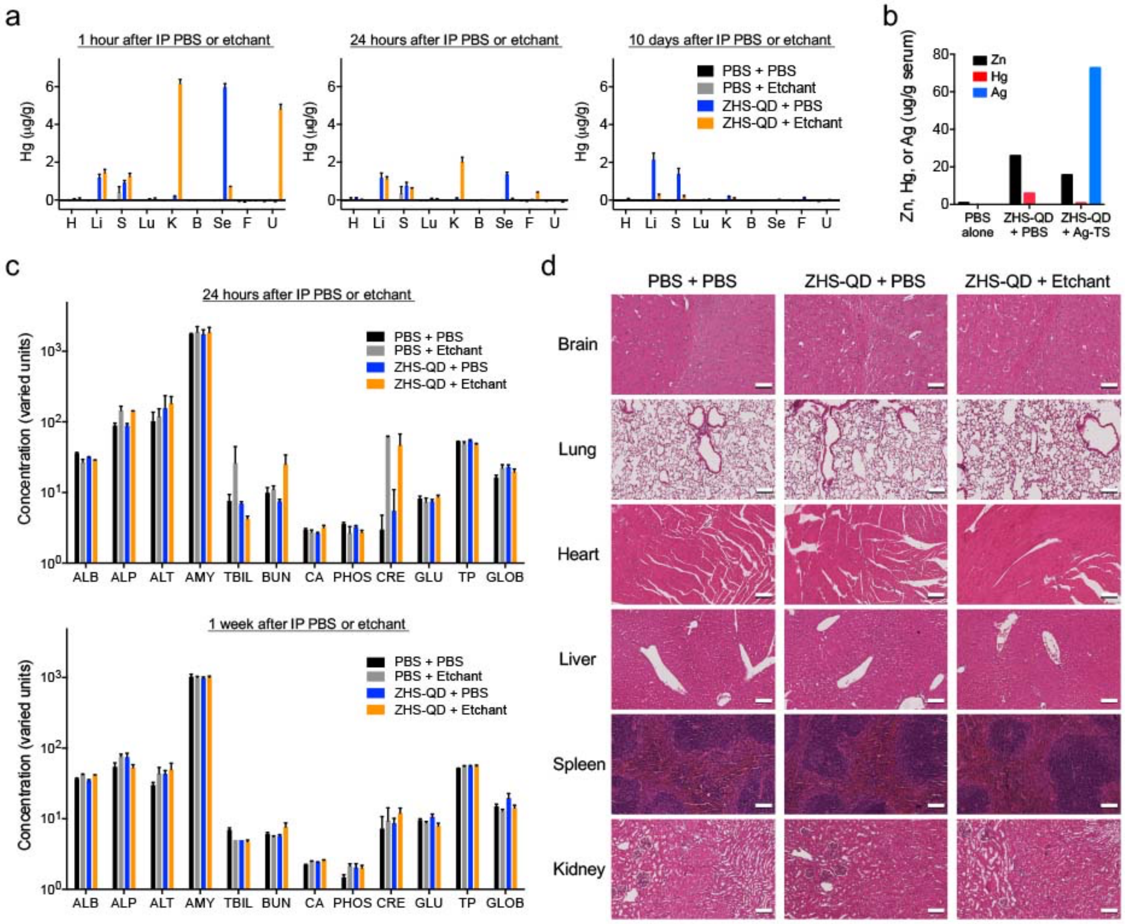

Supplementary Figure 3. ***In vivo*** **clearance and toxicity of the etchable ZHS-QD system.** Mice were intravenously injected with PBS or ZHS-QDs followed 30 min later by intraperitoneal (IP) injection of PBS or etchant (1x Ag-TS). The samples were collected at indicated time points after the IP injections. (**a**) Hg content measured by ICP-OES in tissue, serum (Se), feces (F) and urine (U) samples. H, heart; Li, liver; S, spleen; Lu, lung; K, kidney; B, brain. n = 3 per group. (**b**) Elemental analysis with ICP-OES of ZHS-QDs isolated with Amicon Ultra-0.5 ml centrifugal filters (NMWL: 10K) from the serum of mice 1 h after IP injection of PBS or etchant. (**c**) Plasma biochemical assays performed in mice 24 h (upper panel) and 1 week (lower panel) after IP injection of PBS or etchant. Renal and liver functions were analyzed. n = 6 per group. ALB, albumin (g L^{-1}); ALP, alkaline phosphatase (units L^{-1}); ALT, alanine transaminase (units L^{-1}); AMY, amylase (units L^{-1}); TBIL, total bilirubin (μM); BUN, blood urea nitrogen (mM); CA, calcium (mM); PHOS, phosphorus (mM); CRE, creatinine (μM); GLU, glucose (mM); TP, total protein (g L^{-1}); GLOB, globulin (g L^{-1}). Statistics, two-way ANOVA; error bars, SEM; None of the comparisons between the 4 groups were statistically significant. (**d**) H&E staining results of organs collected from mice 1 week after IP injection of PBS or etchant. Scale bars, 100 μm.

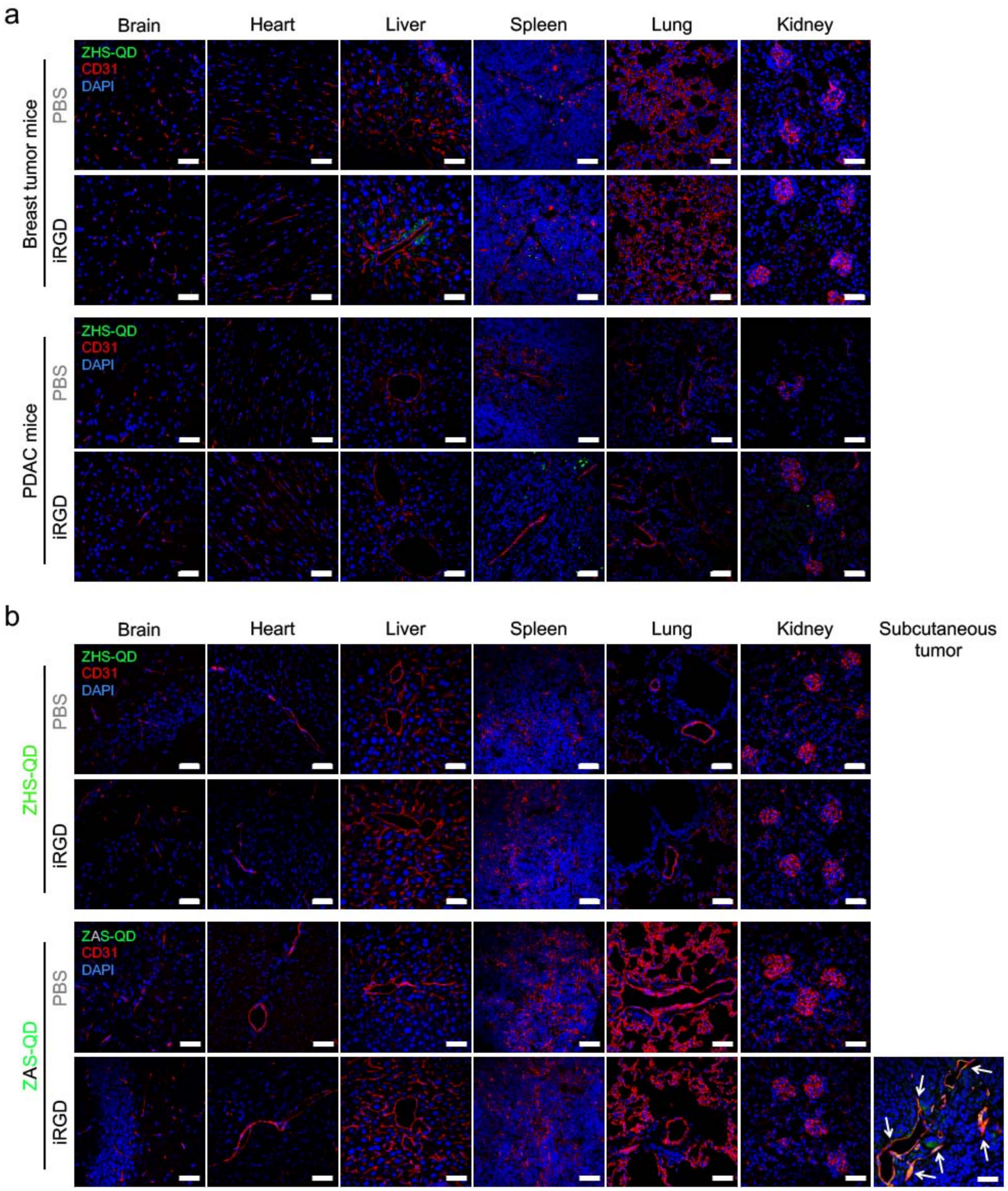

Supplementary Figure 4. QD biodistribution in control tissues. Confocal micrographs of tissues collected from mice bearing orthotopic MCF10CA1a human breast (**a**, upper panels) or KRAS-Ink murine PDAC (**a,** lower panels) tumors, which received PBS or iRGD followed by ZHS-QDs and Ag-TS etchant, and mice bearing MKN45P-luc peritoneal tumors, which received PBS or iRGD with ZHS-QDs (**b,** upper panels) or Z**A**S-QDs (**b,** lower panels) and subsequent Ag-TS. Arrows, blood vessels positive for QDs. n = 3 per group. Blue, DAPI; red, CD31; green, QDs; scale bars, 50 μm.

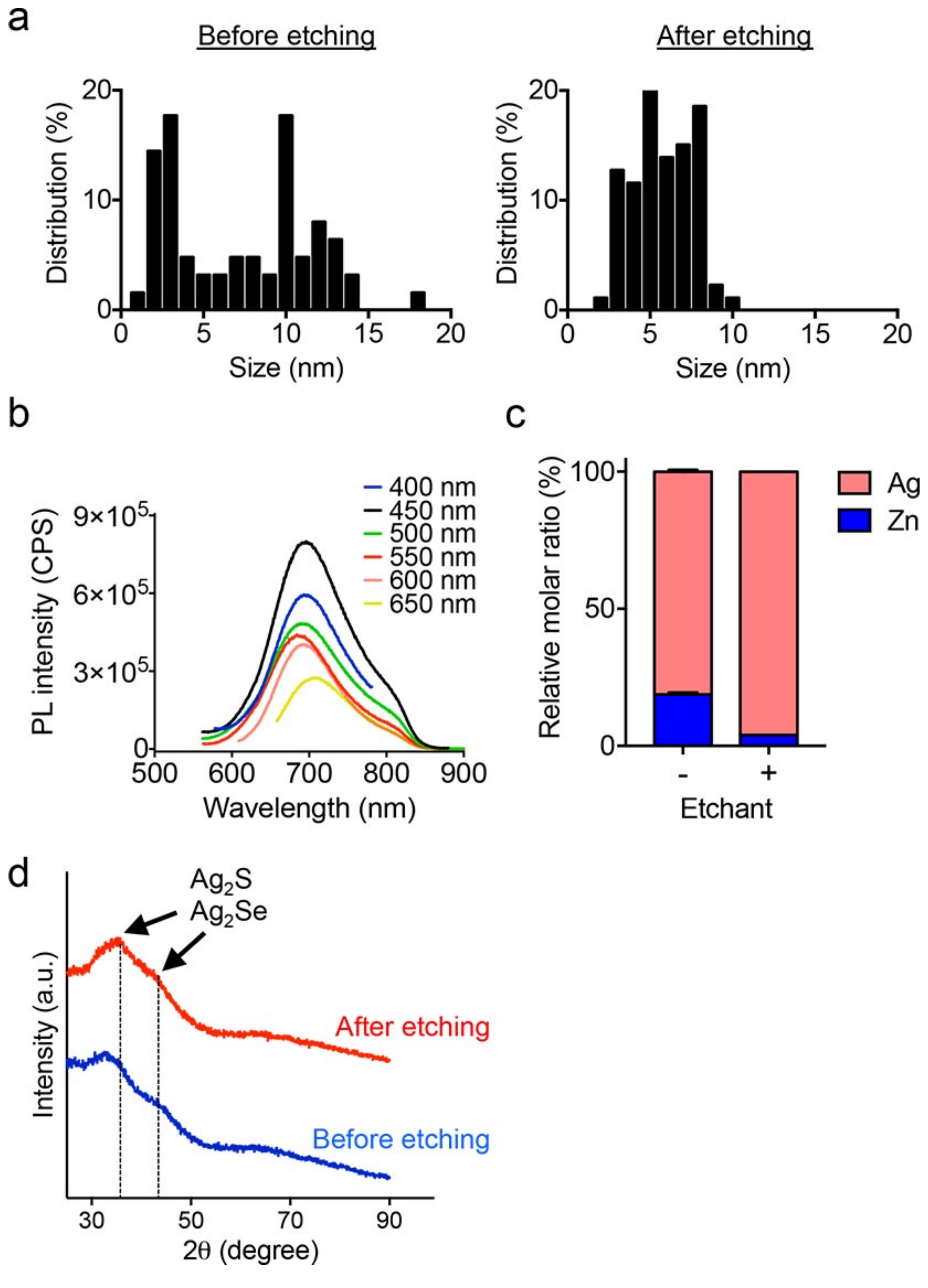

Supplementary Figure 5. Additional characteristics of Z<u>A**</u>S-QDs.** (**a, c, d**) TEM-based size distribution analysis (a), elemental analysis with ICP-OES (c), and crystal structure analysis with XRD (n = 3 per group) (d) of Z**A**S-QDs before and after Ag-TS treatment. In (d), the emergence of a peak at a higher diffraction angle after Ag-TS treatment suggests an increase in the amount of the corresponding elements (Ag_2S and Ag_2Se). (**b**) PL spectra at various excitation wavelengths.

Supplementary Table 1. EDS analysis of ZHS-QDs and ZAS-QDs before and after etching.

Samples[a]	Elemental composition (atomic %)[b]						
	C	O	Zn	Hg	Ag	S	Se
ZHS	69.5 ± 4.9	24.6 ± 5.6	3.0 ± 0.4	0.2 ± 0.0	0.0 ± 0.0	1.3 ± 0.1	1.4 ± 0.2
ZHS, etched	57.1 ± 0.4	31.6 ± 2.3	1.9 ± 0.2	0.0 ± 0.0	4.5 ± 1.1	2.5 ± 0.3	2.3 ± 0.4
ZAS	53.9 ± 9.0	22.9 ± 0.6	3.9 ± 1.6	N/A	6.6 ± 2.3	10.5 ± 3.7	2.2 ± 0.9
ZAS, etched	46.5 ± 5.6	26.7 ± 0.8	1.9 ± 0.4	N/A	11.1 ± 2.4	11.8 ± 1.9	2.0 ± 0.4

[a] All samples were washed thoroughly with H_2O to remove free ions, lyophilized, and subjected for EDS measurement. [b] Average values (mean ± standard deviation) from 3 different areas of each sample under scanning electron microscope observation. N/A, not applicable.

Supplementary Table 2. Summary of the properties of ZHS-QDs and ZAS-QDs.

QDs	Size (nm)		Emission peak (nm)	Molar extinction coefficient (M^{-1} cm^{-1})[a]		Quantum yield
	Before etching	After etching		Before etching	After etching	
ZHS	6.6 ± 2.3	6.0 ± 1.9	685	773.9	18155.2	12%
ZAS	6.8 ± 4.3	5.2 ± 1.8	708	2770.8	13863.7	2%

[a] Molar extinction coefficient (M^{-1} cm^{-1}) was determined at 450 nm in regards to zinc concentration in each QD sample.
